# Augmenting Biogas Process Modeling by Resolving Intracellular Metabolic Activity

**DOI:** 10.3389/fmicb.2019.01095

**Published:** 2019-05-17

**Authors:** Sören Weinrich, Sabine Koch, Fabian Bonk, Denny Popp, Dirk Benndorf, Steffen Klamt, Florian Centler

**Affiliations:** ^1^Biochemical Conversion Department, Deutsches Biomasseforschungszentrum gGmbH, Leipzig, Germany; ^2^Max Planck Institute for Dynamics of Complex Technical Systems, Magdeburg, Germany; ^3^Department of Environmental Microbiology, UFZ – Helmholtz Centre for Environmental Research, Leipzig, Germany; ^4^Bioprocess Engineering, Otto von Guericke University, Magdeburg, Germany

**Keywords:** anaerobic digestion, process modeling, ADM1, flux-balance-analysis, microbial community modeling, metabolic pathways

## Abstract

The process of anaerobic digestion in which waste biomass is transformed to methane by complex microbial communities has been modeled for more than 16 years by parametric gray box approaches that simplify process biology and do not resolve intracellular microbial activity. Information on such activity, however, has become available in unprecedented detail by recent experimental advances in metatranscriptomics and metaproteomics. The inclusion of such data could lead to more powerful process models of anaerobic digestion that more faithfully represent the activity of microbial communities. We augmented the Anaerobic Digestion Model No. 1 (ADM1) as the standard kinetic model of anaerobic digestion by coupling it to Flux-Balance-Analysis (FBA) models of methanogenic species. Steady-state results of coupled models are comparable to standard ADM1 simulations if the energy demand for non-growth associated maintenance (NGAM) is chosen adequately. When changing a constant feed of maize silage from continuous to pulsed feeding, the final average methane production remains very similar for both standard and coupled models, while both the initial response of the methanogenic population at the onset of pulsed feeding as well as its dynamics between pulses deviates considerably. In contrast to ADM1, the coupled models deliver predictions of up to 1,000s of intracellular metabolic fluxes per species, describing intracellular metabolic pathway activity in much higher detail. Furthermore, yield coefficients which need to be specified in ADM1 are no longer required as they are implicitly encoded in the topology of the species’ metabolic network. We show the feasibility of augmenting ADM1, an ordinary differential equation-based model for simulating biogas production, by FBA models implementing individual steps of anaerobic digestion. While cellular maintenance is introduced as a new parameter, the total number of parameters is reduced as yield coefficients no longer need to be specified. The coupled models provide detailed predictions on intracellular activity of microbial species which are compatible with experimental data on enzyme synthesis activity or abundance as obtained by metatranscriptomics or metaproteomics. By providing predictions of intracellular fluxes of individual community members, the presented approach advances the simulation of microbial community driven processes and provides a direct link to validation by state-of-the-art experimental techniques.

## Introduction

Anaerobic digestion is a naturally occurring process driven by a microbial community which is harnessed in biogas plants to convert organic waste material to methane and CO_2_. Being a suitable building block in a renewable energy landscape, biogas production is a popular topic in research and development (Lora Grando et al., 2017). The elucidation of the four sequential steps of anaerobic digestion – hydrolysis, acidogenesis, acetogenesis, and methanogenesis – has allowed for the development of mathematical models that describe the full process. Anaerobic Digestion Model No. 1 (ADM1) is one of the most prominent models and has been in use for more than 16 years (Batstone et al., 2002). The model captures the four process steps of anaerobic digestion by a set of ordinary differential equations (ODEs). Biochemical conversions are expressed as first order kinetics for hydrolysis, and as Monod-type kinetics with additional inhibitory terms for the remaining steps. Microbial activity is resolved on the functional level, with seven state variables indicating the abundance of sugar degraders, amino acid degraders, long chain fatty acid degraders, valerate and butyrate degraders, propionate degraders, and acetoclastic and hydrogenotrophic methanogens. While attempts have been made to include microbial diversity on individual process steps in ADM1 (Ramirez et al., 2009), the inclusion of individual species with their unique metabolic potential has not been attempted before. As experimental data on the genomic level becomes more and more readily available, models are desirable that can take advantage of such data (Kreft et al., 2017). While ADM1 is able to model reactor performance and other abiotic data well, it’s predictions regarding the microbial community, for example total biomass, are less certain. Including microbial community data is hence expected to improve the performance of current models drastically (Lauwers et al., 2013). For sequenced species with annotated genomes, constraint-based techniques are today a standard tool to predict a species’ phenotype from its genotype (Lewis et al., 2012). In particular, Flux-Balance-Analysis (FBA) can be used to predict a population’s growth rate, cross-membrane compound fluxes as well as intracellular metabolic flux distribution, given the metabolic network as defined by the totality of its enzymatic repertoire. These predictions rely on the assumption that intracellular metabolites are at steady state, i.e., their production rates match their consumption rates, and that the cell orchestrates its metabolic flux distribution in order to maximize its growth rate. In dynamic FBA, the steady-state assumption is restricted to consecutive short time intervals, so that dynamic trajectories can be simulated as with regular ODE based models (Antoniewicz, 2013). The FBA approach has been successfully coupled to reactive transport models, increasing model predictive power by reducing the need for empirical calibration (Scheibe et al., 2009). Such models provide quantitative predictions of intracellular activity which are compatible with measured OMICS data targeting transcription activity of enzyme-encoding genes, or enzyme abundance directly. Experimental data can either serve as a benchmark, or can be used to refine the model (Reed, 2012). Dynamic FBA modeling provides predictions on the temporal evolution of up to 1,000s of enzymatically catalyzed metabolic fluxes per species if genome-scale metabolic network models are employed. Such models become available for a steadily increasing number of microbial species (King et al., 2016; Magnúsdóttir et al., 2016). In this work, we evaluate whether biogas process modeling can benefit from these model advancements by coupling selected FBA models of methanogenic archaea of varying complexity to ADM1. For this purpose, we replace the acetoclastic and/or hydrogenotrophic methanogenesis pathways in ADM1 by FBA models of *Methanosarcina barkeri* and *Methanococcus maripaludis* and compare both steady-state simulation results and a pulsed feeding scenario.

## Materials and Methods

To improve the accuracy of representing microbial activity in anaerobic digestion modeling, we couple FBA models of individual methanogenic species to ADM1 where they replace the Monod-type kinetic ODE description of acetoclastic and/or hydrogenotrophic methanogenesis.

### Mass-Based ADM1 Implementation

To depict basic process behavior during anaerobic degradation of organic materials, the established model ADM1 (Batstone et al., 2002) was implemented in Matlab and prepared for coupling with FBA-based modeling. Due to its application in water science and practice, the fundamental reference unit of the standard ADM1 is chemical oxygen demand (COD). Thus, concentrations of individual components are given by their respective COD. However, during degradation of complex particulate materials such as lignocellulosic biomass (e.g., agricultural waste or energy crops) the COD is not a suitable reference unit for substrate characterization. Most often individual conversion factors based on the theoretical COD of relevant components have been applied to map analytical results to respective input and output variables of ADM1 (Lübken et al., 2007; Wichern et al., 2009; Koch et al., 2010).

To reveal stoichiometric degradation pathways and enable consistent application of ADM1 during anaerobic degradation of particulate materials, the COD-based model structure was rewritten for its direct application in biogas technology (Weinrich, 2017). Based on the theoretical COD or respective mol weight of each component (Huete et al., 2006), the fundamental model structure was transformed entirely to a mass-based reference unit. All affected model parameters describing substrate degradation, microbial growth or inhibition were converted accordingly. To ensure a closed nitrogen and carbon balance in all 19 processes, additional terms were added to the respective differential equations for inorganic nitrogen S_IN_ and carbon S_IC_. Further changes include the calculation of pH inhibition based on Hill functions as well as the gas flow calculation due to an overpressure in the head space. Default parameter values as presented in the standard ADM1 (Batstone et al., 2002) were used and translated to a mass-based reference unit. The detailed description of model derivation as well as the complete set of differential, algebraic equations, and individual parameter values are presented in Weinrich (2017).

### Flux-Balance-Analysis Based Modeling

Flux-Balance-Analysis is an established method which enables the prediction of the metabolic phenotype from a species’ genotype (O’Brien et al., 2015). Briefly, the enzymatic potential as encoded on a species’ genome is used to define its metabolic network as the sum of all enzymatic metabolite conversion steps it is capable of. The key assumption of FBA is that intracellular metabolites are at steady state. This requires for all metabolites that production rates match consumption rates so that metabolite concentrations do not change. Mathematically, this can be expressed by the equation *Nv* = 0, in which *N* is the stoichiometric matrix encoding the metabolic network and *v* is a flux vector which assigns a particular flux to each individual reaction of the network. Besides enzymatic reactions, *N* additionally includes pseudo reactions that detail the transport of metabolites across the cell membrane and a biomass reaction that encodes the composition of the species’ biomass. While solving the steady-state equation delivers a whole range of possible flux distributions, a single solution can be derived as the model prediction if additionally assuming that the cell orchestrates its metabolic fluxes such that growth is maximized. This is done by solving a linear programming problem consisting of the steady-state equation, potentially further flux restrictions, and maximizing the flux for the biomass reaction. To implement the non-growth associated energy demand for maintenance, a reaction transforming ATP to ADP is typically included in the network and fixed to a particular flux given in mmol ATP/gDW/h. As optimization results of the linear programming problem are not necessarily unique, and additionally assuming that the cell tries to maximize growth with minimal enzymatic effort, we use the parsimonious variant of FBA (Lewis et al., 2010). This requires a two-step procedure in which, after the maximal growth rate is identified, a flux distribution is sought which achieves this growth rate by the smallest possible fluxes, i.e., for which |*v*| is minimized. We implemented this approach in Matlab and used CellNetAnalyzer (von Kamp et al., 2017) for individual FBA simulations.

### Replacing Microbial Growth in ADM1 by FBA

Generally, microbial growth and decay is described in ADM1 by a Monod-type equation with additional inhibitory terms:

$$\frac{dX}{dt}\;\text{=}\;\text{μ}(S)\;\cdot \;X\;\cdot \;I\;-\;k_{d}\;\cdot \;X,\;\;\;\;\;\;\;\;\;\;\;\;\;\;\;(1)$$

where *X* represents microbial biomass concentration (g/L), μ(*S*) is the substrate concentration dependent specific growth rate defined as $\text{μ}\;=\;{\text{μ}}_{max}\;\cdot \;\frac{S}{K_{S}+S}$, with the maximal specific growth rate μ*_max_* (1/d), substrate concentration *S* (g/L) and half-saturation constant *K_S_* (g/L). *I* is the product of inhibitory terms *I_n_* having values ranging from 0 (complete inhibition) to 1 (no inhibition), and *k_d_* is the specific biomass decay rate (1/d). ADM1 considers methanogenesis via the acetoclastic and the hydrogenotrophic pathway, with each pathway being described by an ODE of the form of Eq. 1. For the acetoclastic methanogens, the growth-limiting substrate in Eq. 1 is acetate, while it is hydrogen for the hyrogenotrophic methanogens. In our coupled model, we model one or both pathways by FBA models of specific methanogens. For this, we need to specify the maximal substrate uptake rate *v_maxUptake_*, which depends on the substrate concentration *S* in the reactor:

$$v_{maxUptake}\;\text{=}\;v_{max}\;\cdot \;\frac{S}{K_{S}+S}\;\cdot \;I,\;\;\;\;\;\;\;\;\;\;\;(2)$$

with maximal uptake rate *v_max_* (mmol/gDW/h). Note that we use the same inhibitory term(s) *I* as in Eq. 1 to restrict substrate uptake to replicate ADM1’s implementation of growth inhibition. Using *v_maxUptake_*, the FBA model is then used to predict the current specific growth rate μ*_FBA_*, and microbial growth can then be modeled as:

$$\frac{dX}{dt}\;\text{=}\;{\text{μ}}_{FBA}\;(S,I)\;\cdot \;X\;-\;k_{d}\;\cdot \;X.\;\;\;\;\;\;\;\;\;\;\;\;\;\;\;(3)$$

**Figure 1:**
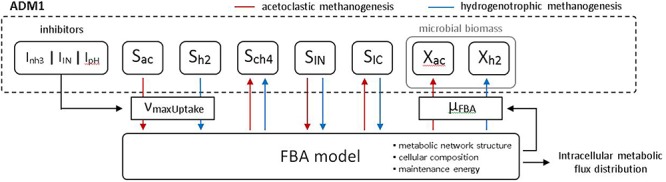
Coupling concept of the augmented model. ADM1 state variables for acetate (S_ac_) and hydrogen (S_h2_) are used to define maximal allowable uptake fluxes for the methanogenic FBA model, taking into account ADM1 inhibitory processes. The FBA model predicts the current specific growth rate μ_FBA_ which is used to update respective biomass state variables in ADM1. Predicted uptake and release fluxes are likewise used to update ADM1 state variables. In addition, the FBA model provides predictions for all intracellular fluxes.

For parameterizing Eq. 2, we use the same numeric values for *K_S_* as in Eq. 1 and select *v_max_* so that in absence of substrate limitations and inhibitory processes, the maximal growth rate becomes identical to the value used in ADM1, that is μ*_FBA_* = μ*_max_*. For describing the impact of microbial activity on substrates and products in the standard ADM1, Eq. 1 is multiplied by a yield factor. This is not necessary in the coupled model for those steps which are replaced by FBA models, as these directly predict uptake and excretion rates of substrates and products, only requiring the conversion of these fluxes given in mmol/gDW/h to g/L/d, including a multiplication with the current biomass *X* of the respective microbial population. For acetoclastic methanogenesis, we couple methane and acetate directly. The compounds CO_2_ (and additionally HCO_3_ if present in the FBA model) and NH_4_ are linked to ADM1’s state variables for inorganic carbon and inorganic nitrogen. To implement the coupled model, we use the direct approach in which calls to the FBA solver are placed in the function which is used by the numerical solver to evaluate the right-hand side of the ODE system. The Matlab solver ode15s (variable order algorithm based on numerical differentiation formulas) is used to numerically solve the coupled model. The fundamental coupling concept and transfer of state variable values between ADM1 and FBA models is illustrated in Figure 1.

### FBA Models and Coupling Variants

Flux-Balance-Analysis models of two methanogens, *M. barkeri* and *M. maripaludis*, were used to simulate methanogenesis in ADM1 (Table 1). We used small-scale FBA models derived from Koch et al. (2016). Additionally, we employed a genome-scale model for *M. barkeri* (Gonnerman et al., 2013), referred to as “*M. barkeri* (GS)” from here on, to identify possible benefits of such comprehensive models. *M. barkeri* can perform both acetoclastic and hydrogenotrophic methanogenesis, while *M. maripaludis* can only produce methane from hydrogen. Maximal substrate uptake rates (*v_max_* in **Eq. 2**) were set for FBA growth prediction to match ADM1’s μ*_max_* values, as described above (Table 2). We implemented a series of model variants in which either only one methanogenic pathway was simulated by a FBA model, or both simultaneously. In the latter case, either *M. barkeri* was assumed to be able to use both pathways at the same time, or the *M. barkeri* model was restricted to acetoclastic methanogenesis and accompanied by *M. maripaludis* performing hydrogenotrophic methanogenesis (Table 3).

**Table 1:** Models of methanogenic species used.

Species	Available pathways	Number of reactions	Number of internal metabolites	Reference
*Methanosarcina barkeri*	acetoclastic and hydrogenotrophic	103	96	Koch et al., 2016
*Methanosarcina barkeri* (genome-scale)	acetoclastic and hydrogenotrophic	816	642	Gonnerman et al., 2013
*Methanococcus maripaludis*	hydrogenotrophic	102	95	Koch et al., 2016

**Table 2:** Pathway- and model-specific kinetic uptake values.

Pathway	*v_max_* (mmol/gDW/h)		μ*_max_* (1/d)	*K_m_* (g/L)
	computed for NGAM^1^			
	set to 0.5 mmol			
	ATP/gDW/h			
Acetoclastic	*M. barkeri*	1.82	0.4	0.1408
	*M. barkeri* (GS)	3.66		
Hydrogenotrophic	*M. barkeri*	29.73	2.1	8.82 × 10^-7^
	*M. barkeri* (GS)	65.60		
	*M. maripaludis*	70.39		

*Values for μ_*max*_ and K_*m*_ are taken from Weinrich and Nelles (2015) and v_*max*_ was computed, so that FBA growth predictions match μ_*max*_. ^*1*^non-growth associated maintenance*.

**Table 3:** Model coupling variants.

Model variant	acetoclastic pathway	hydrogenotrophic pathway	Number of FBA models
1	*M. barkeri*	ADM1	1
2	*M. barkeri* (GS)	ADM1	1
3	ADM1	*M. barkeri*	1
4	ADM1	*M. barkeri* (GS)	1
5	ADM1	*M. maripaludis*	1
6	*M. barkeri*	*M. barkeri*	1
7	*M. barkeri* (GS)	*M. barkeri* (GS)	1
8	*M. barkeri*	*M. maripaludis*	2

## Input Characterization

Steady-state and dynamic simulations were based on substrate characteristics and an experimental setup for anaerobic mono-digestion of maize silage. In a previous study, fermentation of different energy crops (maize, grain, and sugar beet silage) was implemented in a continuously operated 45 L (V_liq_ = 37 L) laboratory-scale reactor at mesophilic temperatures at a hydraulic retention time of 185 d (q_in_ = 0.2 L/d) (Weinrich and Nelles, 2015). Phase 1 in that study referred to mono-digestion of maize silage and serves as the reference scenario for our simulations. The reactor input is characterized by concentrations of degradable carbohydrate, protein, and lipids, as well as the respective organic acids contained in the utilized maize silage (Table 4, converted from Table 6 in Weinrich and Nelles, 2015).

**Table 4:** Input composition during continuous anaerobic digestion of maize silage, taken from Weinrich and Nelles (2015).

ADM1 model component	Input concentration (g/L)
X_ch_	202.4
X_pr_	16.3
X_li_	12.0
S_va_	0.2
S_bu_	0.56
S_pro_	0.64
S_ac_	2.6
S_IN_	1.5

To ensure sufficient nitrogen supply the input concentration for inorganic nitrogen was set to 1.5 g per L substrate added. All remaining input concentrations of additional state variables considered in ADM1 (including anions, cations, and microbial biomasses) were set to zero.

## Results

### Comparing Growth and Yields

**Figure 2:**
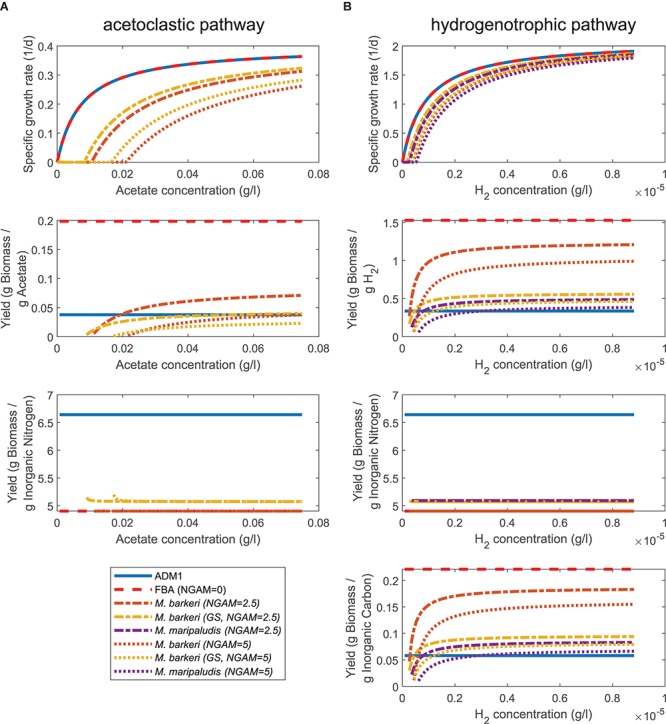
Comparing growth and yield predictions under varying substrate concentrations for ADM1 and FBA models. **(A)** Acetoclastic methanogenesis pathway consuming acetate and inorganic nitrogen (NH_4_). **(B)** Hydrogenotrophic methanogenesis pathway consuming hydrogen, inorganic nitrogen (NH_4_) and inorganic carbon (CO_2_, HCO_3_^-^).

We first compared the prediction of specific growth rates and yields under varying substrate concentrations using either ADM1’s ODE for acetoclastic and hydrogenotrophic methanogenesis or the respective FBA models. If setting the non-growth associated maintenance (NGAM) demand to zero, the FBA-predicted specific growth rate matched ADM1’s prediction (Figure 2) as the maximal uptake *v_max_* for FBA simulations was chosen accordingly (see section “Materials and Methods”). If NGAM demand is increased to 2.5 and 5 mmol ATP/gDW/h, a larger fraction of the consumed substrate needs to be transformed to energy and is no longer available for biomass synthesis. As a consequence, predicted specific growth rates become smaller as NGAM demand is increased. Additionally, growth becomes infeasible below a critical substrate concentration. As a fraction of consumed substrate is used for fulfilling cellular maintenance requirements, the observed biomass yield *Y_X/S_* becomes a function of the growth rate.

For slow growth close to the minimal substrate concentration threshold, almost all substrate is used for maintenance leading to small values for *Y_X/S_*. At high substrate concentrations and fast growth, in contrast, the yield approaches the constant yield assumed in ADM1 and, depending on chosen values for the maintenance requirement, surpasses this value (Figure 2). This was also the case for the yield with respect to inorganic carbon for the hydrogenotrophic pathway, but not for the yield with respect to inorganic nitrogen for both pathways. For inorganic nitrogen, yields were constant along the tested substrate concentration range and in value smaller than in ADM1 simulations. The different FBA models of the same species generally agreed well in their predictions regarding specific growth rate and yields.

### Steady-State Behavior

In the next step, we implemented a constant feeding scenario based on the substrate and process characteristics of anaerobic mono-digestion of maize silage (as described in the section “Materials and Methods”). We simulated the coupled models until the steady state was reached. To evaluate the impact of the chosen NGAM demand, we systematically varied the demand between 0 and 5 mmol ATP/gDW/h and compared resulting steady states with the standard ADM1 model predictions.

**Figure 3:**
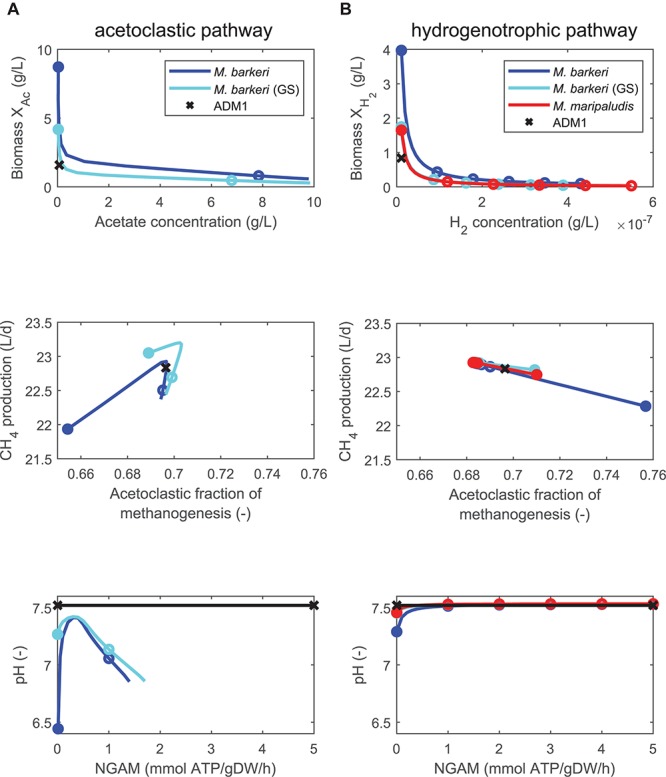
Comparing steady-state model predictions of standard ADM1 (x) and coupled models in which the non-growth associated maintenance (NGAM) demand is varied between 0 (closed symbols) and 5 mmol ATP/gDW/h (open symbols mark increments of 1), using continuously fed maize silage as substrate. Two pathways contribute to methanogenesis: **(A)** Only the acetoclastic pathway is replaced by FBA models of *M. barkeri*, either by a minimal or a genome-scale model (GS). **(B)** Only the hydrogenotrophic pathway is replaced by FBA models of *M. barkeri* [same variants as for **(A)**] or *M. maripaludis*.

First, we consider model variants 1 to 5 in which only one methanogenic pathway was replaced by a FBA model (Table 3). If NGAM demand was set to zero, the coupled models predicted higher biomass concentrations and lower substrate concentrations than the standard ADM1 model (Figure 3). As NGAM demand is increased more substrate must be channeled toward providing the required maintenance energy, resulting in smaller biomass concentrations and higher substrate concentrations in the reactor. Increasing NGAM demand, predictions first approach ADM1 results, but then lead to lower predictions of biomass and higher predictions of substrates than ADM1. For the acetoclastic pathway, higher acetate concentrations lead to lower pH values and acidification. This in turn inhibits methanogenesis and can lead to a reactor breakdown. Due to this dependency, NGAM values could not be increased beyond a value of 1.4 (1.7) mmol ATP/gDW/h for acetoclastic methanogenesis in the (genome-scale) FBA model of *M. barkeri*, as from there on, acidification with a subsequent process breakdown was predicted by the coupled model. The general tendency to first approach ADM1 predictions, but then overshooting or undershooting them as NGAM demand is increased was also true for the predicted methane production rate, pH values, and the contribution of both methanogenesis pathways toward methane production (Figure 3).

**Figure 4:**
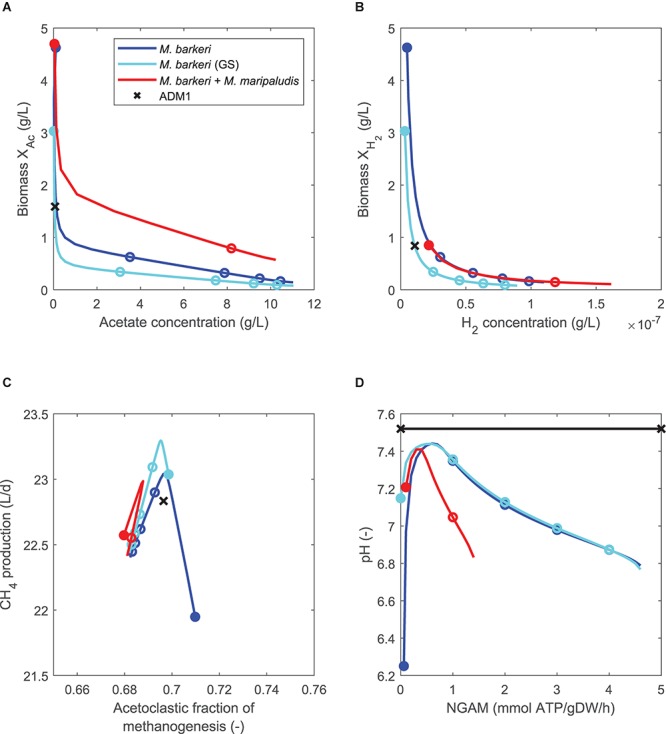
Comparing steady-state model predictions of standard ADM1 (x) and coupled models in which the non-growth associated maintenance (NGAM) demand is varied between 0 (closed symbols) and 5 mmol ATP/gDW/h (open symbols mark increments of 1), using continuously fed maize silage as substrate. Both methanogenesis pathways are replaced by either one FBA model of *M. barkeri* [minimal or genome-scale (GS) model], or by two pathway-specific models. **(A)** Comparing biomass of acetoclastic population (X_AC_) and acetate concentration. **(B)** Comparing biomass of hydrogenotrophic population (X_H2_) and hydrogen concentration. Note that for single FBA model coupling variants, biomass values are identical to **(A)**. **(C)** Comparing methane production rate and pathway-specific contribution to methanogenesis. **(D)** Comparing resulting pH as NGAM is increased.

For model variants in which both pathways are replaced either by one (model variants 6 and 7, Table 3) or two FBA models (model variant 8), this behavior was equally observed (Figure 4). However, while all steady-state predictions of all three model variants generally followed similar trends, the model combining pathway-specific FBA models of *M. barkeri* and *M. maripaludis* showed some deviations. First, NGAM values could not be increased beyond 1.4 mmol ATP/gDW/h as acidification led to reactor breakdowns for higher values, similar to when this *M. barkeri* model was used to replace the acetoclastic pathway before (Figure 3A). Second, in comparison to the other model variants, higher biomass concentrations for the acetoclastic, but lower biomass concentrations for the hydrogenotrophic population are predicted for the considered NGAM range (Figure 4A,B). Contrary to this shift, the hydrogenotrophic pathway was predicted to contribute slightly more to overall methanogenesis than in the other model variants (up to 3%-points for NGAM = 0, Figure 4C).

### Dynamic Simulation

**Figure 5:**
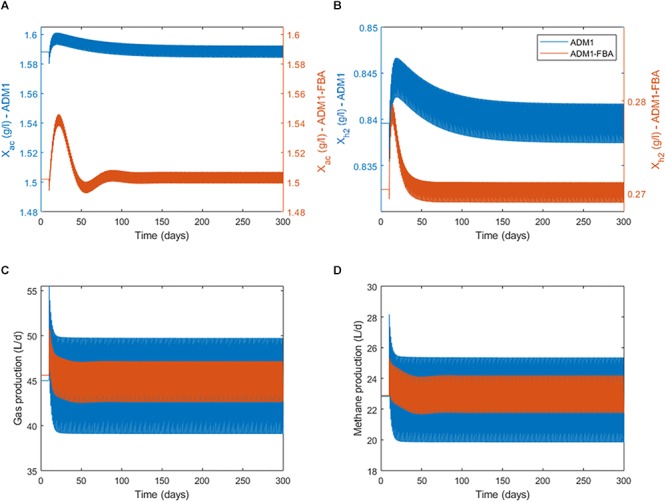
Switching at day 10 from continuous feeding to a daily feeding pulse of 1 h providing the same amount of substrate and comparing standard ADM1 to the coupled model variant 8. Bands result from daily oscillations not resolvable here; see Figure 6 for daily dynamics. **(A)** Biomass concentration of the acetoclastic population. **(B)** Biomass concentration of the hydrogenotrophic population. Note the secondary y-axes scaling. **(C)** Process performance in terms of total gas production. **(D)** Process performance in terms of methane production.

Next we considered model variant 8 in which both methanogenesis pathways are modeled by distinct FBA models in a dynamic simulation. We chose for both models a NGAM value of 0.5 mmol ATP/gDW/h. We started the simulation with continuous feeding, using the previously established steady states as initial conditions. After 10 days of continuous feeding, we switched the feeding to a pulsed feeding in which a daily feeding pulse of 1 h provides the same substrate to the reactor as the continuous feeding distributed over a full day, so that the organic loading rate (and hydraulic retention time) remains unchanged. Once the feeding regime is switched, a regular oscillatory behavior results after a transient phase (Figure 5). Mean process performance in terms of produced gas volume and methane, and biomass concentrations of both methanogenic populations during pulsed feeding are very close to the previous steady state under continuous feeding for both the standard ADM1 model and the coupled model (Table 5). This is not surprising as the total substrate input remains the same.

**Table 5:** Steady-state simulation results for the dynamic simulation of continuous and pulsed feeding, both featuring identical daily substrate input rates.

	ADM1		Coupled model	
			(variant 8,	
			NGAM = 0.5 mmol	
			ATP/gDW/h)	
State variable (unit)	Continuous feeding	Pulsed feeding^1^	Continuous feeding	Pulsed feeding^1^
*X_ac_* (g/L)	1.59	1.59	1.50	1.50
*X_h2_* (g/L)	0.84	0.84	0.27	0.27
Gas production (L/d)	45.01	45.04	45.60	45.63
Methane production (L/d)	22.83	22.85	22.90	22.91
Fraction of acetoclastic methanogenesis (-)	0.70	0.70	0.69	0.69

^1^*calculated as the average over the last 50 days of the simulation*.

Anaerobic Digestion Model No. 1’s and the coupled model’s predictions regarding process performance agree very well, but differ considerably regarding biomass concentrations of both methanogenic populations and their dynamics (Figure 5A,B). The coupled model predicts the biomass of the acetoclastic population to be 5.7% smaller, and the biomass of the hydrogenotrophic population to be 67.9% smaller than the standard ADM1 model. Interestingly, this shift in microbial biomass does not lead to a shift in the contribution of both pathways to methanogenesis: both models predict 69–70% of methanogenesis to be provided by the acetoclastic pathway (Table 5). Regarding the adaptation dynamics to pulsed feeding, the coupled model predicts a faster response of the biomass. Regular oscillatory behavior is reached after approximately 100 days for the acetoclastic and after 40 days for the hydrogenotrophic population. This is much faster compared with the 140 days required in the standard ADM1 model (Figure 5).

**Figure 6:**
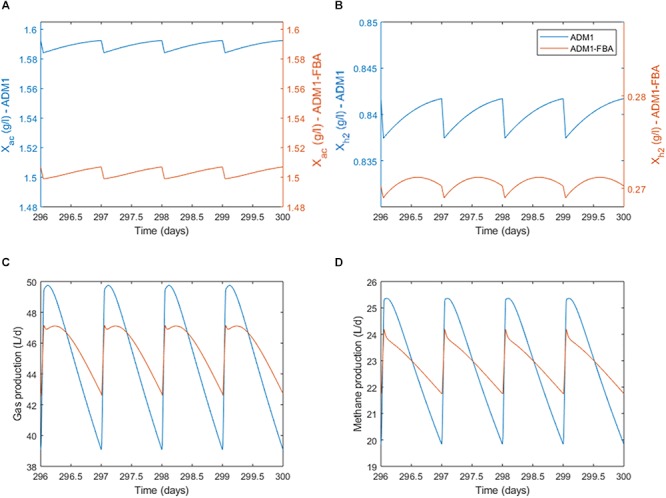
Daily dynamics for the pulsed feeding regime. **(A)** Biomass concentration of the acetoclastic population. **(B)** Biomass concentration of the hydrogenotrophic population. Note the secondary y-axes scaling. **(C)** Process performance in terms of total gas production. **(D)** Process performance in terms of methane production.

**Figure 7:**
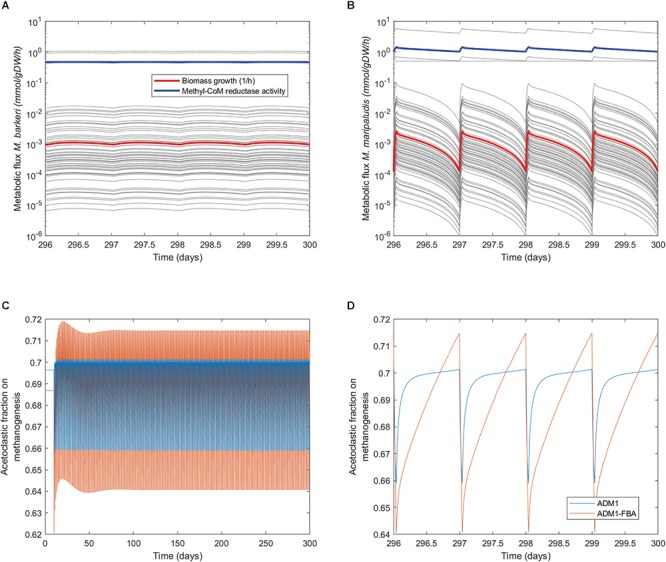
Daily dynamics of methanogens’ growth, intracellular fluxes, and pathway contribution to methanogenesis. **(A,B)** Dynamics of specific growth rates and metabolic fluxes of the methanogenic FBA models, highlighting flux through methyl-coenzyme M reductase as the last step in methanogenesis. **(C)** Dynamics of the contribution of the acetoclastic and hydrogenotrophic pathway to methanogenesis. **(D)** Daily dynamics of the contribution of both pathways to methanogenesis.

When inspecting the daily dynamics for the pulsed feeding regime in detail, we find that for both the standard ADM1 and the coupled model the acetoclastic population shows an almost linear growth which is periodically interrupted by the feeding pulse (Figure 6A). As a fraction of the reactor content is replaced during feeding, biomass is lost during the feeding pulse. The specific growth rate for *M. barkeri* as predicted by the coupled model remains fairly constant (Figure 7A). For the hydrogenotrophic *M. maripaludis* population, the coupled model predicts that even before the pulse event, its biomass starts to decrease midway between pulses as cellular decay surpasses growth (Figure 6B). This is in contrast to standard ADM1’s predictions in which biomass increases throughout the non-feeding period. Regarding process performance, the coupled model predicts a lower amplitude for both gas and methane production, and less steep gradients in the non-feeding period (Figure 6C,D). Overall, the coupled model predicts a greater difference in activity between both methanogenic pathways. While growth rate and with it all intracellular fluxes remain almost constant for the acetoclastic *M. barkeri* population, dynamics of the hydrogenotrophic *M. maripaludis* population is characterized by a sharp increase in growth rate and intracellular fluxes during the feeding pulse, followed by an accelerating decrease in activity during the non-feeding period (Figure 7A,B). These stronger differences in the activity of both pathways results in a larger amplitude of the contribution of both pathways to total methanogenesis (Figure 7C,D). Generally, methanogenesis is mainly attributed to acetoclastic methanogens (with 69–70% on average). Hydrogenotrophic methanogenesis has its highest share at the onset of the feeding pulse, where growth and activity of *M. maripaludis* increases sharply. This share slowly shifts as its growth rate decreases while growth rate and activity of *M. barkeri* remains almost constant.

## Discussion

### Opening the Gray Box: Making Models OMICS-Data-Ready

After 16 years of process-based modeling of anaerobic digestion with ADM1, we here demonstrate the feasibility of replacing individual process steps in ADM1 – here the two methanogenesis pathways – by microbial species-centric FBA models detailing intracellular metabolic activity in down to genome-scale detail. Tracking the dynamics of complex microbial communities in terms of compositional changes over time has become feasible by cultivation-independent techniques focusing on next-generation-sequencing [amplicon sequencing targeting the 16S rRNA gene (Pace, 1997)] or single-cell based methods (Liu et al., 2018). Although the absolute quantification of species-specific cell numbers or biomass in complex communities remains a challenge (Bonk et al., 2018a), relative compositional data have become readily available. To make use of such data, mathematical models need to contain state variables which refer to these populations. ADM1 focuses on process steps which are mathematically described by ODEs and which are carried out not by a single species but typically by a community of varying diversity, depending on the process step. ADM1 has been extended before to include a number of hypothetic species which catalyze the same process step but with different kinetic parameters to account for this diversity (Ramirez et al., 2009). This approach provided insights into the effect of diversity on reactor performance and response to perturbations in terms of resistance and resilience. However, as this approach sticks to ADM1’s ODE formulation to describe metabolic activity, many more difficult to measure parameters were necessary in the model. These had to be chosen randomly, making this approach difficult to fit to experimental data on microbial community dynamics. Instead of increasing diversity, our model focusses on improving the description of biochemical activity for individual process steps. In this first step, we replace the ODE formulation subsuming the activity of all species performing a process step by a FBA model of one representative species. In the next step, additional competing species can be included as FBA models to capture the diversity as in the model by Ramirez et al. (2009). Individual FBA models detail how substrate and other compounds taken up from the environment by a particular microbial species are transformed into biomass, maintenance energy, and secreted products by the species’ metabolic network. Enzymatically catalyzed reactions are the building block of these networks, and models provide quantitative predictions for all intracellular fluxes. Such data can be compared to measured enzyme activity obtained by metatranscriptomics or metaproteomics. Taking the opposite route, such experimental data can also be used to constrain the model (Reed, 2012), although the correlation between transcriptomic data, proteomic data, and actual enzyme activity is still under debate (Haider and Pal, 2013). Nevertheless, this enhanced compatibility with state-of-the-art experimental techniques is a great asset of the presented coupled model.

### Model Coupling and Comparison

Coupling FBA models to ADM1 using the direct approach proved to be easy to implement and feasible in terms of the additionally required computational demand. The complexity and stiffness of ADM1’s ODEs likely make the static or dynamic optimization approach, as alternatives to the direct approach, infeasible due to their excessive computational demand (Mahadevan et al., 2002). The coupled model inherits the flexible parametrization available in ADM1, for example allowing for the easy implementation of changing compositions of the reactor input and dynamic feeding regimes. Simulation results regarding overall process performance agreed well between the standard ADM1 and the novel coupled models. Differences were observed regarding the biomass concentrations and their dynamics for the microbial species now simulated by FBA models. Both methanogenic populations of *M. barkeri* and *M. maripaludis* had lower predicted biomass concentrations in the coupled model. Taken together with the unaltered prediction of methane production, the novel model hence predicts a higher per cell activity of the respective populations. Under a pulsed feeding scenario, the reference ADM1 model predicted identical trajectories of the biomass concentration for both methanogens, while the coupled model predicted the growth dynamics of the hydrogenotrophic population to fluctuate more, leading to stronger shifts of the contribution of both pathways, yet overall leading to lower amplitudes in gas and methane production. Non-continuous feeding had been shown before to favor more robust microbial communities (De Vrieze et al., 2013; Bonk et al., 2018b). Exploring the limits of flexible feeding regimes is additionally an important aspect that needs more consideration within the context of the flexibilization of biogas production. This would allow to offset the fluctuating output of other renewable, weather-dependent energy supplies such as wind and solar energy (Mauky et al., 2017).

Yields, which are specified as constant parameters in ADM1, do no longer need to be specified in the coupled model, where they are determined by the available substrate, the current demand for cellular maintenance, and the topology of the metabolic network. Yields become a model output, allowing for a more realistic incorporation of the non-constant relationship between yield and growth rate (Lipson, 2015) in the coupled models. Maximization of microbial growth was used as the optimization criterion during FBA computations. Except for a fixed flux constraint for considering the non-growth associated maintenance demand, no other constraints were imposed on internal fluxes, so that predicted flux distributions were also optimal with respect to biomass yield. This would not be the case for overflow metabolism situations. To consider such cases, protein allocation can additionally be considered and biomass yield instead of growth rate can be optimized for, requiring more sophisticated techniques (Adadi et al., 2012; Klamt et al., 2018).

To model *M. barkeri*, we used both a minimal (103 reactions) and a genome-scale FBA model (816 reactions). Simulation results agreed very well qualitatively (Figure 2–4), and only minor quantitative differences were observed, for example a minimal acetate concentration of 17 mg/L, allowing for growth for the genome-scale model vs. 22 mg/L for the minimal model. In light of these minor differences, the minimal model seems to capture the essential growth features of *M. barkeri* very well and is sufficient to model this organism as part of an anaerobic digestion microbial community.

### Non-growth Associated Maintenance

For the coupled model, we observed predicted reactor breakdowns for NGAM values beyond 1.4 mmol ATP/gDW/h, when simulated under constant conditions. For the dynamic simulation, we selected a value of 0.5 mmol ATP/gDW/h as this value led to steady-state predictions close to those of the standard ADM1. While higher values have been estimated before, including 0.9 mmol ATP/gDW/h for *M. maripaludis*, and 3.6 mmol ATP/gDW/h for acetate converting *M. barkeri* [collected in (Koch et al. (2016)], maintenance energies have also been reported to be lower than theoretical predictions under methanogenic conditions (Scholten and Conrad, 2000). A combination of experimental and modeling approaches has recently suggested even smaller values of below 0.116 mmol ATP/gDW/h for short hydraulic retention times, likely being applicable to also longer hydraulic retention times (Bonk et al., 2019). Fitting with this observation, maintenance demands in a binary propionate utilizing syntrophic methanogenic culture were experimentally determined to be 0.14 mmol ATP/gDW/h for *Syntrophobacter fumaroxidans* and 0.025 mmol ATP/gDW/h for *Methanospirillum hungatei* (Hamilton et al., 2015). Under slow growing conditions such as in anaerobic digestion, care must hence be taken when selecting NGAM values, as they strongly impact predicted community composition (Koch et al., 2016).

### Choice of Species to Model

To add species-specific state variables to ADM1, we started by replacing ODE descriptions of both methanogenic pathways by species-specific FBA models. Methanogenesis is the archaea-driven last of four steps in anaerobic digestion. It is associated with a low microbial diversity, which increases toward the first steps of the process (Campanaro et al., 2016). As only few currently known archaea are capable of methanogenesis and as they are well-described, the choice of methanogenesis as our first target was both natural and the most straight-forward. We only considered one species per pathway, while in typical biogas microbiomes, more than one methanogenic species will compete for acetate and hydrogen. Our coupled model can easily be extended to include such competition. Somewhat more challenging is the replacement of the other three steps preceding methanogenesis in anaerobic digestion. Not only is the microbial diversity higher in those steps, but also the functional roles of only few species have been elucidated. For acetogenesis during which volatile fatty acids are transformed to acetate for example, only three syntrophic propionate oxidizers and three syntrophic butyrate oxidizers have been described (Worm et al., 2014). A further bottleneck is the availability of FBA models. For example, up to date no FBA model for any of the three known butyrate oxidizers is publicly available. This requires additional efforts in generating the required models, although current computational pipelines brought model development times from years down to weeks (Latendresse et al., 2012). And even for not yet cultivated species, growing genomic resources focusing on anaerobic digestion hold the promise to reconstruct at least draft models for such species (Campanaro et al., 2016), ultimately allowing for FBA-based coupled models to capture the natural diversity of anaerobic digestion. We selected the methanogenic species in this study based on their relevance in anaerobic digestion and the availability of respective FBA models. Once models become available for methanogens often found in biogas reactors including for example *Methanoculleus marisnigri* and *Methanosaeta concilii*, it will be possible to include exactly those species in the model, which have been found to be dominant in the anaerobic digestion process to be simulated, for example based on 16S rRNA gene- and/or *mcrA*-based community analysis. Such tailor-made models offer the most faithful representation of the actual microbial community at hand and are expected to surpass current modeling approaches in their predictive power for simulating industrial biogas processes.

## Conclusion

We are only at the beginning to understand the complex interplay at work in microbial communities in natural or engineered systems. Community systems biology and quantitative modeling are instrumental in advancing our understanding (Zengler and Palsson, 2012; Hanemaaijer et al., 2015). Here, for anaerobic digestion which is harnessed in biogas reactors to produce methane from waste streams, we provide a first step to switch from process-based gray box models to species-based community models. As these models provide flux predictions on the enzyme level down to the genome-scale, they provide a convenient common reference to which multi OMICS data can be related to. Likewise, such data can be used to refine the model by constraining fluxes to observed ranges. Integrating multi OMICS data with modeling is a promising strategy to elucidate microbial interactions in complex communities (Zuñiga et al., 2017). While FBA models have been employed before to determine optimal community compositions at steady state (Koch et al., 2016), we here expand this approach to the dynamic situation. Our coupled models provide a convenient tool to interpret time series data from operational biogas plants, to explore theoretically possible maximal yields and process efficiency, to identify early warning signals indicating looming reactor breakdowns and to test intervention strategies to avoid costly reactor breakdowns. To make optimal use of microbial community driven processes, the need for active management has been recognized (Carballa et al., 2015). In this context, mechanistic community models resolving intracellular activity are a crucial component, which prospectively could be integrated into a model-based online monitoring and control scheme.

## Data Availability

The raw data supporting the conclusions of this manuscript will be made available by the authors, without undue reservation, to any qualified researcher.

## Author Contributions

FC introduced the initial idea and coordinated the study. SW, SKo, FB, DP, SKl, and FC designed the study. SKo, DB, and SKl developed metabolic models, SW implemented the ADM1 model. FC implemented the metabolic flux modeling component, performed simulations and analyzed the data. All authors read and approved the final manuscript.

## Contribution to the Field Statement

Anaerobic digestion is a naturally occurring process that is harnessed in biotechnological settings to produce biogas as a flexible and renewable energy source. It is a multi-step process which is brought about by a complex microbial community. Modeling this process to simulate industrial-scale biogas plants has been done for many years by the model ADM1, which is based on ordinary differential equations. In this approach, the activity of microbes is considered by their Input–Output behavior, that is, how much substrate is how fast transformed into products and cellular growth. Metabolic activity within the cell is not considered. However, the intracellular activity of microbial cells has been elucidated to a level at which networks of thousands of enzymatic reactions and metabolites detail, how substrates are converted to products and biomass. Furthermore, current experimental techniques deliver data on the activity on individual enzymes and pathways part of these networks. To make use of such data, we combine ADM1 with the Flux-Balance-Analysis modeling technique, which works on the level of species-specific metabolic networks. Our coupled models are a first step toward models that more faithfully represent microbial activity on the single species-level in complex community simulations. Such models can take advantage of current experimental techniques and hence promise to surpass current models in their predictive power.

## Conflict of Interest Statement

The authors declare that the research was conducted in the absence of any commercial or financial relationships that could be construed as a potential conflict of interest.
